# Seven-Day Nonbismuth Containing Quadruple Therapy Could Achieve a Grade “A” Success Rate for First-Line *Helicobacter pylori* Eradication

**DOI:** 10.1155/2015/623732

**Published:** 2015-05-19

**Authors:** Wei-Chen Tai, Chih-Ming Liang, Chen-Hsiang Lee, Chien-Hua Chiu, Ming-Luen Hu, Lung-Sheng Lu, Yuan-Hung Kuo, Chung-Mou Kuo, Yi-Hao Yen, Chung-Huang Kuo, Shue-Shian Chiou, Keng-Liang Wu, Yi-Chun Chiu, Tsung-Hui Hu, Seng-Kee Chuah

**Affiliations:** ^1^Division of Hepatogastroenterology, Department of Internal Medicine, Kaohsiung Chang Gung Memorial Hospital and Chang Gung University College of Medicine, 123 Ta-Pei Road, Niao-Sung District, Kaohsiung City 833, Taiwan; ^2^Division of Infectious Diseases, Department of Internal Medicine, Kaohsiung Chang Gung Memorial Hospital and Chang Gung University College of Medicine, 123 Ta-Pei Road, Niao-Sung District, Kaohsiung City 833, Taiwan; ^3^Division of Internal Medicine, Department of Internal Medicine, Kaohsiung Chang Gung Memorial Hospital and Chang Gung University College of Medicine, 123 Ta-Pei Road, Niao-Sung District, Kaohsiung City 833, Taiwan

## Abstract

This prospective study was to assess the efficacy of nonbismuth containing quadruple therapy as first-line* H. pylori *treatment and to determine the clinical factors influencing patient outcome. We enrolled 200* H. pylori*-infected naïve patients. They were prescribed either a 7-day nonbismuth containing quadruple therapy group (EACM, esomeprazole 40 mg twice daily, amoxicillin 1 g twice daily, metronidazole 500 mg twice daily, and clarithromycin 500 mg twice daily) or a 7-day standard triple therapy group (EAC, esomeprazole 40 mg twice daily, amoxicillin 1 g twice daily, and clarithromycin 500 mg twice daily). Follow-up studies to assess treatment responses were carried out 8 weeks later. The eradication rates attained by EACM and EAC groups were 95.6% (95% confidence interval [CI] = 89.4%–98.3%) and 79.3% (95% CI = 70%–86.4%) in the per-protocol analysis (*P* < 0.001) and 88% (95% CI = 80.2%–93.0%) and 73% (95% I = 63.6%–80.3%) in the intention-to-treat analysis (*P* = 0.007). Clarithromycin resistance, metronidazole resistance, and dual clarithromycin and metronidazole resistances were the clinical factors influencing* H. pylori *eradication in EACM group. Clarithromycin resistance and dual clarithromycin and metronidazole resistances were the influential factor for EAC treatment. In conclusion, the results suggest that 7-day nonbismuth containing quadruple therapy could achieve a grade “A” report card for first-line* H. pylori *treatment.

## 1. Introduction

The Maastricht IV/Florence-Consensus Report recommended that the standard triple therapy should now be avoided in areas where clarithromycin resistance is high (>15–20%) [1]. The reported local primary resistance rate to clarithromycin in Taiwan ranged from 6 to 18% over time [2–7]. However, Chen et al. recently reported the primary resistance rates of metronidazole (52.8% versus 47.7%) and clarithromycin (13.9% versus 22.7%) in patients who lived in urban and rural areas of eastern Taiwan [8]. The bismuth containing quadruple therapy with 10-day duration could be the alternative first-line treatment, especially in areas with a high prevalence of clarithromycin and metronidazole resistance, because of its ability to overcome metronidazole resistance and achieve an eradication rate > 90% [9–11]. Other alternatives include sequential therapy, concomitant therapy, and hybrid therapy, which provide > or close to 90% eradication rates even in areas with high rates of clarithromycin and metronidazole resistance. However, there were inconsistent reports for the eradication rates of sequential therapy. For instance, Wu et al. [2] and Tsay et al. [12] both reported >90% intention-to-treat (ITT) and per-protocol (PP) eradication rates in Taiwan. In Korea, the reported PP eradication rate of sequential therapy was 85.7% without at least grade “B” report card in 2008 [13]. This year, there were two publications from Korea and China which reported a decrease in 10-day sequential therapy which was only 72.1% by ITT and 78.4% by PP analysis for the Korean report [14] and 72.1% by ITT and 76.5% by PP analysis for the Chinese report [15]. The bottom line is that the inconsistent results may imply that the efficacy of sequential therapy varies in different countries and may have declined over time. It might not be a perfect option in the first-line* H. pylori* eradication anymore, particularly in areas where clarithromycin resistance is high (>15–20%).

One of the other alternatives includes hybrid therapy, which could provide >95% eradication rate even in areas with high rates of clarithromycin and metronidazole resistance [16]. Hsu et al. reported near 100% PP eradication rates for 14-day hybrid therapy [16]. It has two phases: dual therapy with a PPI (standard dose, b.i.d.) and amoxicillin (1 g, b.i.d.) for 7 days, followed by a nonbismuth quadruple therapy consisting of PPI (standard dose, b.i.d.), amoxicillin (1 g, b.i.d.), clarithromycin (500 mg, b.i.d.), and metronidazole (500 mg, b.i.d.) for further 7 days. The benefit of the extended duration of amoxicillin administration is to further reduce the bacterial load to improve the eradication rate but then again it involves very complex regimens [16]. However, recently published reports from Korea and Iran reported eradication rates of 85.9% and 92.9% [17, 18]. It may need more studies to prove its efficacies in different countries and taking into account the local antibiotics resistance to metronidazole and clarithromycin. Besides, the complexity and lengthy duration of the prescription may be the disadvantage.

Concomitant therapy consists of a PPI (standard dose, b.i.d.) combined with clarithromycin (500 mg, b.i.d.), amoxicillin (1 g, b.i.d.), and metronidazole (500 mg, b.i.d.), prescribed all together at the same time which is more convenient than sequential therapy and hybrid therapy because of the shorter duration of treatment and less complex drug administration. As a matter of fact, the concomitant therapy was more convenient and achieves equally efficient eradication rates and is with consistent results over years. Studies with 10-day concomitant therapy achieved an efficacy of > or close to 90% for* H. pylori* eradication rates [2, 19–24]. The current study explored the influential role of 7-day nonbismuth containing quadruple therapy for first-line* H. pylori* eradication to determine which clinical factors influence patient outcome.

## 2. Materials and Methods

### 2.1. Patients

From August 2012 to March 2014, a total of 200* H. pylori*-infected naïve patients were enrolled. All patients were at least 18 years of age and had received endoscope examinations that showed peptic ulcers or gastritis. They were randomly prescribed either a 7-day nonbismuth containing quadruple therapy group (EACM, esomeprazole 40 mg twice daily, amoxicillin 1 g twice daily, metronidazole 500 mg twice daily, and clarithromycin 500 mg twice daily for 7 days) or a 7-day standard triple therapy group (EAC, esomeprazole 40 mg twice daily, amoxicillin 1 g twice daily, and clarithromycin 500 mg twice daily, for 7 days) by their clinicians.

Criteria for exclusion included the following: (1) previous surgery of the stomach, such as partial gastrectomy; (2) use of antibiotics within the preceding 30 days; (3) regular use of a PPI or bismuth compounds (>3 times per week) in the 30 days before enrollment; (4) presence of serious medical condition(s) precluding participation or endoscopy with biopsy; (5) patients previously treated for* H. pylori* infection; (6) use of concomitant medication(s) known to interact with study medication (simvastatin was permitted); (7) presence of Zollinger-Ellison syndrome; (8) pregnancy or lactation; (9) allergy to any medication in the study; (10) contraindication(s) to the use of any of the study drugs; (11) participating in any clinical trial within the last 30 days; (12) unwillingness to abstain from alcoholic beverages; and (13) patients taking other medications including antipsychotics, and chronic nonsteroidal anti-inflammatory drugs were also excluded.

This study was approved by both the Institutional Review Board and the Ethics Committee of Chang Gung Memorial Hospital (IRB-101-0674A3). All patients provided their written informed consent before undergoing endoscopic interventions.

### 2.2. Outcomes and Follow-Up

The primary endpoint was the successful eradication of* H. pylori*. We also analyzed antibiotic susceptibility. The confirmation of* H. pylori* eradication failure was defined as positive results both for the rapid urease test and by histology after first-line eradication therapy. All registered patients were followed up at week 2 to assess drug compliance and adverse effects after they finished the medication regimens. Drug compliance was assessed via pill counts. Poor compliance was defined as failure to finish 80% of all medication due to adverse effects [25–28]. These patients underwent either an endoscopy or a urea breath test 8 weeks later. We also performed a back-up urea breath test on all participants to avoid any false-negative results.

### 2.3. Questionnaire

The questionnaire contained questions regarding personal history of smoking and alcohol drinking. The questionnaire was locally derived and not a validated or previously published quality of life questionnaire. Quality of life was not assessed. Smokers were defined as those who consumed more than 1 pack of cigarettes a week, and drinkers were those who drank more than 1 cup of alcoholic beverage per day. The adverse events evaluated included abdominal pain, diarrhea, constipation, dizziness, taste perversion, headache, anorexia, nausea, vomiting, and skin rash. Those who considered those symptoms disturbed their daily life were defined to have major adverse effects. Those who experienced these symptoms but did not consider them a disturbance to their daily life were defined to have minor adverse effects.

### 2.4. Culture and Antimicrobial Resistance

One antral gastric specimen and one corpus biopsy specimen were obtained for* H. pylori* isolation using previously described culture methods [29]. The biopsy specimens were cultured on plates containing* Brucella* chocolate agar with 7% sheep blood and incubated for 4-5 days under microaerobic conditions. The minimal inhibitory concentration (MIC) was determined by the agar dilution test.* H. pylori* strains with MIC values ≥0.5, ≥1, ≥1, ≥4, and ≥8 mg/L were considered to be the resistant breakpoints for amoxicillin, clarithromycin, levofloxacin, tetracycline, and metronidazole, respectively [2, 3, 16, 25].

### 2.5. Randomization

A computer-generated randomization list was used to generate a “random sequence.” We used a method combining blocking and stratified randomization to ensure a close balance of the numbers and patients' characteristics in each group. We set separate randomization within each of two groups of participants. We then set a block of every 10 participants. A computer-generated randomization list was drawn up by the statistician and given to the physician for randomization. Doctors determined patients' suitability to be enrolled in this study and allocated the next available number on entry into the trial. The code was revealed to the researchers once recruitment, data collection, and laboratory analyses were complete. An independent research assistant generated the computerized random number sequence. The sequence was concealed in an opaque envelope until the intervention was assigned. After the written informed consents were obtained from the participants, an independent research assistant assigned the therapies according to the treatment allocations kept in the envelopes. Each patient collected the medications on the same day from the pharmacy department in our hospital.

### 2.6. Statistical Analysis

The primary outcome variables were the eradication rate, presence of adverse events, and level of patient compliance. Using the SPSS program (Statistical Package for the Social Sciences version 18, Chicago, IL, USA), Chi-square tests with or without Yates' correction for continuity and Fisher's exact tests were used when appropriate to compare the major outcomes between groups. Eradication rates were analyzed by both ITT and per-protocol (PP) approaches. ITT analysis included all assigned patients who had taken at least one dose of the study medication. Patients whose infection status was unknown following treatment were considered treatment failures for the purposes of the ITT analysis. The PP analysis excluded patients with unknown* H. pylori* status following therapy and those with major protocol violations. A *P* value <0.05 was considered statistically significant. To determine the independent factors that affected treatment response, the clinical and bacterial parameters were analyzed by univariate and multivariate analyses.

## 3. Results

**Figure 1:**
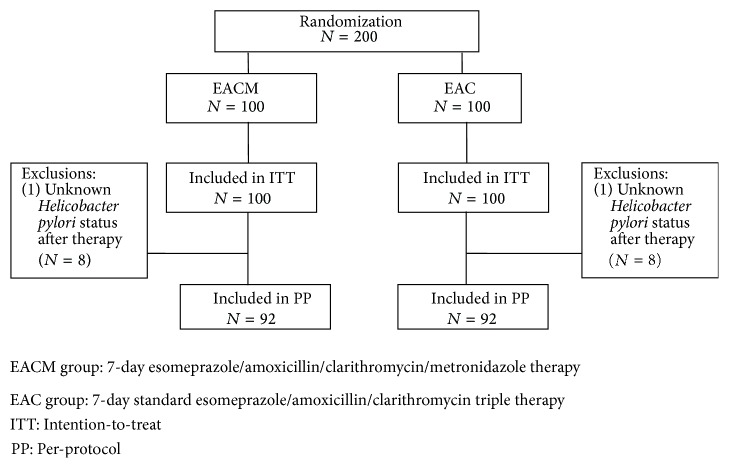
Disposition of patients.

Figure 1 shows patient flowchart, according to the CONSORT statement advice. A total of 200 patients with positive* H. pylori* were recruited into the study and were randomly assigned to receive EACM and EAC therapy. The two treatment groups were matched with respect to baseline demographic data, clinical characteristics, and antibiotic resistance (Table 1). A total of 16 patients were excluded from the PP analysis (8 in each group), because of being lost to follow-up, resulting in 92 in the PP study for each of EACM and EAC groups. The eradication rates in the EAC and EACM groups are detailed in Table 2. They were 95.6% (95% confidence interval [CI] = 89.4–98.3%) and 79.3% (95% CI = 70%–86.7%), respectively, in the PP analysis (*P* < 0.001); 88% (95% CI = 80.2%–93%) and 73% (95% CI = 63.6%–80.3%), respectively, in the ITT analysis.

### 3.1. Adverse Events and Complications

The adverse event rates were 30.4% (28/92) in EACM group and 16.3% (15/92) in EAC group, *P* = 0.024 (Table 3). These adverse events included abdominal pain, constipation, diarrhea, dizziness, headache, and nausea/vomiting; there were more patients in the EACM group experiencing nausea sensation than the EAC group (30.4% versus 16.3%, *P* = 0.024). However, these were mild and did not markedly disturb the patients' daily activities and importantly both groups had good drug compliances (100% in all of them).

### 3.2. Antibiotic Resistance

Samples from 90 patients were cultured for* H. pylori*, and the positive culture rate was 75.6% (68/90). The results of the* H. pylori* eradication rate for different subgroups susceptible to amoxicillin, clarythromycin, and metronidazole for both EAC and EACM patients are foundin Table 4.

### 3.3. Factors Influencing the Efficacy of Anti-*H. pylori* Therapies

Univariate analysis for factors that influence the efficacy was shown in Table 4. Clarithromycin resistances (CLA-R) were significant factors in both the EACM group (*P* = 0.002) and the EAC group (*P* < 0.001). Metronidazole resistance (MET-R) is the risk factors for eradication failure in the EACM group (*P* = 0.009) but not the EAC group. However, dual resistances to both antibiotics were the risk factors for eradication failure in both EACM and EAC groups (*P* < 0.001).

## 4. Discussion

Our data confirm that the previously reported efficacy of using concomitant therapy achieves an efficacy of > or close to 90% for* H. pylori* eradication rates but we achieved a grade “A” PP result (95.6%) with 7-day regimen instead of 10-day one [2, 21–24]. These findings merit the recommendations of concomitant therapy as the optimal formula in addition to the advantage of being far more easy and convenient for patients to follow than the sequential and hybrid therapies.

Possible explanations for the discrepancies in the reports for both sequential and hybrid therapies as first-line* H. pylori* eradication regimens are different antibiotic resistances of* H. pylori* strains and different treatment durations. Besides the regional variations in eradication efficacies, sequential and hybrid therapy are much more complex in terms of medication requirements because the patients need to switch drugs during the treatment course. This might result in reducing patient compliance and physician preference to prescribe the regimen [16–18, 30]. Therefore, concomitant therapy might be a relatively optimal treatment option in terms of convenience and consistent efficacies among the three. Nevertheless, the inevitable problematic issue is that CLA-R and MET-R could be crucial in the efficacy of* H. pylori* eradication [31].

Currently, the decrease in the first-line* H. pylori* eradication rates in standard 7-day triple therapy is relevant to the progressively increasing CLA-R resistance rates in many parts of the world, particularly those areas with the CLA-R above 20% [30]. The current study showed eradication rates of 73% in ITT and 79.3% in the PP analysis in EAC group. Univariate and multivariate analyses of our data identified CLA-R as a factor that reduced the efficacy of concomitant therapy. None of our 19 patients with CLA-R assigned to EAC were eradicated and the same occurred to the other six patients with dual resistance (CLA-R and MET-R) as shown in Table 4. This was the same for EACM group. Two out of four CLA-R patients in the EACM group were eradicated (50%) while the eradication rates dropped to 33.3% when dual resistance was present. These findings implied that dual resistance could be a major factor affecting the outcome of both EACM and EAC patients. The bottom line is that the prevalence of dual resistance in our study was only 13.2% among the PP population (9/68) and the low number of patients makes the possibility of a type II error likely. For instance, the majority of dual resistances that had treatment failure in EAC arm were due to clarithromycin resistance as demonstrated by the fact that none of the 19 clarithromycin resistance strains were successfully eradicated while 4/10 of metronidazole resistance strains were still successfully eradicated under the EAC arm. Therefore, we should be careful during interpretation of these data. It would be overestimated by drawing conclusion that dual clarithromycin and metronidazole resistance influences the treatment outcome of EAC arm. The effect could be likely due to clarithromycin resistance alone.

When bismuth quadruple therapy is not available, nonbismuth containing quadruple therapy (concomitant therapy) is recommended in high prevalence of CLA-R areas by the Maastricht IV Consensus Report [1]. As mentioned earlier in this text, the reported local primary resistance rate to clarithromycin in Taiwan is increasing over time [2–8]. It was therefore suggested that standard seven-day triple therapy is not suitable for patients in Eastern Taiwan because the* H. pylori* eradication rates were only 57.5% by ITT analysis and 61.8% by PP analysis. This is the same in current study as we attained only an eradication rate of 79.3% by PP analysis and 73% by ITT analysis. This is the same in current study as we attained only an eradication rate of 79.3% by PP analysis and 73% by ITT analysis. Both these Taiwanese studies, including ours, suggested that the clinical practice for first-line eradication with standard triple therapy should be abandoned. However, for patients who encountered dual resistances, although 7-day EACM eradication rates are higher than EAC group (33.3% versus 0%, *P* = 0.134), it implies that in areas with high prevalence of CLA-R even concomitant therapy may not be suitable to eradicate patients with CLA-R and MET-R resistances. The impact of MET-R on the outcome of concomitant therapy is relatively less marked than CLA-R. Data on* H. pylori* eradication with concomitant therapy in isolated MET-R populations from previously published studies indicate that eradication rates are >85% [2, 23], which is similar to our own findings (72.7% in the EACM group versus 40% in the EAC group).

This study encounters several limitations. Firstly, the sample size is clearly insufficient for a treatment comparison. An adequate sample for reliably detecting clinically significant differences between the two arms would be much larger than the number of patients included in the study. Therefore this study should be considered to be a pilot study that reports preliminary results on the two first-line therapies rather than being treated as a comparative trial. Secondly, the small populations with antibiotic resistance data included in this study have impeded the evaluation of the effects of antibiotic resistance on eradication efficacy. Large sample sized, prospective randomized studies are mandatory to precisely interpret the association of antibiotic resistance to the efficacy of concomitant therapy in Taiwan. Up to now, standard triple therapy was still recommended by the Taiwanese National Health Insurance Administration as the first-line empiric regimen but the overall eradication rates have dropped to <80% as shown in current study. We believe that our results will help facilitate the selection of appropriate patients for concomitant therapy in Taiwan because the more significant rise in clarithromycin resistance is unavoidable for Taiwanese in the near future. It is therefore likely that eradication rates will continue to drop.

## 5. Conclusion

In conclusion, the results suggest that a 7-day nonbismuth containing quadruple therapy could achieve a grade “A” report card (>95% eradication rate) for first-line* H. pylori* treatment.

## Figures and Tables

**Table 1:** Demographic data and endoscopic appearances of the two patient groups.

Characteristics	EACM (*n* = 92)	EAC (*n* = 92)	*P* value
Age (year) (mean ± SD)	47.8 ± 11.6	52.8 ± 12.8	0.593
Gender (male/female)	45/47	46/46	0.883
Smoking	14	14	1.000
Alcohol consumption	20	14	0.254
Previous history of peptic ulcer	14	19	0.337
Endoscopic findings			0.964
Gastritis	32	34	
Gastric ulcer	22	22	
Duodenal ulcer	31	28	
Gastric and duodenal ulcer	7	8	

EACM group: 7-day esomeprazole/amoxicillin/clarithromycin/metronidazole therapy; EAC group: 7-day standard esomeprazole/amoxicillin/clarithromycin/triple therapy.

**Table 2:** Major outcomes of eradication therapy.

	Eradication rate		
	EACM (*n* = 92)	EAC (*n* = 92)	*P* value
Intention-to-treat	88% (88/100)	73% (73/100)	0.007
Per-protocol	95.6% (88/92)	79.3% (73/92)	<0.001
Adverse event	30.4% (28/92)	16.3% (15/92)	0.024
Compliance	100% (92/92)	100% (92/92)	1.000

EACM group: 7-day esomeprazole/amoxicillin/clarithromycin/metronidazole therapy; EAC group: 7-day standard esomeprazole/amoxicillin/clarithromycin/triple therapy.

**Table 3:** Adverse events during eradication therapies.

Adverse event	EACM (*n* = 92)	EAC (*n* = 92)	*P* value
Abdominal pain	9 (9.8%)	6 (6.5%)	0.419
Constipation	1 (1.1%)	2 (2.2%)	0.560
Diarrhea	11 (11.9%)	6 (6.5%)	0.203
Dizziness	7 (7.6%)	4 (4.3%)	0.315
Headache	6 (6.5%)	2 (2.2%)	0.148
Nausea/vomiting	10 (10.8%)	2 (2.2%)	0.017
Skin rash	0 (0%)	0 (0%)	—
Taste perversion	0 (0%)	0 (0%)	—

EACM group: 7-day esomeprazole/amoxicillin/clarithromycin/metronidazole therapy; EAC group: 7-day standard esomeprazole/amoxicillin/clarithromycin/triple therapy.

**Table 4:** Univariate analysis of the clinical factors influencing the efficacy of *H. pylori* eradication.

Principle parameter		EACM			EAC		
		Case number	Eradication rate (%)	*P* value	Case number	Eradication rate (%)	*P* value
Age	<60 years	75/78	96.1	0.578	52/64	81.2	0.496
	≥60 years	13/14	92.8		21/28	75.0	
Sex	Female	44/47	93.6	0.328	33/46	71.1	0.071
	Male	44/45	97.8		40/46	86.9	
Smoking	(−)	74/78	94.9	0.386	61/78	78.2	0.523
	(+)	14/14	100.0		12/14	85.7	
Alcohol consumption	(−)	69/72	95.8	0.872	61/78	78.2	0.523
	(+)	19/20	95.0		12/14	85.7	
Previous history of peptic ulcer	(−)	74/78	94.9	0.386	57/73	78.1	0.557
	(+)	14/14	100.0		16/19	84.2	
Compliance	Good	92/92	100.0	—	92/92	100.0	—
	Poor	0	0		0	0	

*H. pylori* culture (*n* = 68)							
Amoxicillin	Susceptible	31/34	91.2	—	15/33	45.4	0.367
	Resistance	0	—		0/1	0	
Clarithromycin	Susceptible	29/30	96.7	0.002	15/15	100	<0.001
	Resistance	2/4	50.0		0/19	0	
Metronidazole	Susceptible	23/23	100	0.009	11/24	45.8	0.755
	Resistance	8/11	72.7		4/10	40.0	
Dual resistance	Absent	30/31	96.8	<0.001	15/28	53.6	<0.001
	Present	1/3	33.3		0/6	0	

EACM group: 7-day esomeprazole/amoxicillin/clarithromycin/metronidazole therapy; EAC group: 7-day standard esomeprazole/amoxicillin/clarithromycin triple therapy.
